# Reduced bone resorption by intake of dietary vitamin D and K from tailor-made Atlantic salmon: a randomized intervention trial

**DOI:** 10.18632/oncotarget.10171

**Published:** 2016-08-14

**Authors:** Ingvild Eide Graff, Jannike Øyen, Marian Kjellevold, Livar Frøyland, Clara Gram Gjesdal, Bjørg Almås, Grethe Rosenlund, Øyvind Lie

**Affiliations:** ^1^ National Institute of Nutrition and Seafood Research (NIFES), Bergen, Norway; ^2^ Department of Rheumatology, Haukeland University Hospital, Bergen, Norway; ^3^ Department of Clinical Science, University of Bergen, Bergen, Norway; ^4^ Hormone Laboratory, Haukeland University Hospital, Bergen, Norway; ^5^ Skretting Aquaculture Research Centre, Stavanger, Norway

**Keywords:** Atlantic salmon, bone health, vitamin D, vitamin K, bone biomarkers, Gerotarget

## Abstract

Suboptimal vitamin D status is common among humans, and might increase bone resorption with subsequent negative effects on bone health. Fatty fish, including Atlantic salmon, is an important dietary vitamin D source. However, due to a considerable change in fish feed composition, the contribution of vitamin D from salmon fillet has been reduced. The main objective was to investigate if intake of vitamin D_3_ enriched salmon or vitamin D_3_ tablets decreased bone biomarkers (urinary N-telopeptides, deoxypyridinoline, serum bone-specific alkaline phosphatase, and osteocalcin) compared to a low vitamin D_3_ intake. The 122 healthy postmenopausal women included in this 12 weeks intervention trial were randomized into four groups: three salmon groups (150 grams/two times/week) and one tablet group (800 IU vitamin D and 1000 mg calcium/day). The salmon groups also received calcium supplements. The salmon had three different vitamin D_3_/vitamin K_1_ combinations: high D_3_+high K_1_, low D_3_+high K_1_, or high D_3_+low K_1_. Increased intake of salmon containing high levels of vitamin D_3_ (0.35-0.38 mg/kg/fillet) and supplements with the same weekly contribution had a positive influence on bone health as measured by bone biomarkers in postmenopausal women. Consequently, an increased level of vitamin D_3_ at least to original level in feed for salmonids will contribute to an improved vitamin D_3_ status and may improve human bone health.

## INTRODUCTION

Proper nutrition plays a crucial role in both the prevention and the treatment of osteoporosis. In addition to calcium, vitamin D from dietary or supplement sources have historically been the major therapeutic focus [1]. Suboptimal vitamin D status is common among humans in general [2, 3]. Fatty fish like Atlantic salmon (*Salmo salar*) is one of the very few natural dietary sources of vitamin D [4]. However, due to a considerable change in fish feed composition with less marine and more plant based ingredients the contribution of vitamin D from salmon fillet has been reduced. Thus, the content of vitamin D in commercial farmed Atlantic salmon is relatively low [4]. Fatty fish have also a naturally high content of long-chain omega-3 polyunsaturated fatty acids (LC-PUFA) which have been suggested to have a role in the prevention of osteoporosis due to several mechanisms, including its altering of the immune function [5, 6].

The prevalence of osteoporosis [7, 8] and osteoporotic fractures [9] are especially high in Norway, and low vitamin D levels have been observed in patients with osteoporosis [1, 10], distal radius fracture [10], and hip fracture [11]. Further, general findings from prospective studies in community-dwelling populations indicate that low concentrations of vitamin D are associated with future fracture risk, and randomized controlled studies shows fracture preventive effects of combined supplement with vitamin D and calcium [12]. In addition, the general elderly population is at risk of vitamin D deficiency [13]. Osteocalcin is produced by osteoblasts and incorporated into the bone matrix. It is released into the circulation from the matrix during bone resorption and, therefore, is considered a marker of bone turnover rather than a specific marker of bone formation [14, 15]. Osteocalcin is carboxylated by the vitamin K-dependent γ carboxyglutamic acid [16] and this process is critical for osteocalcin's ability to bind hydroxyapatite. In humans, the fraction of the circulating osteocalcin that is not γ-carboxylated (GLU/GLA) is used as a biomarker of vitamin K status [17, 18]. Low levels of vitamin K_1_ and K_2_ has been observed in osteoporotic patients [19, 20], and low dietary intake of vitamin K_1_ has been associated with low bone mineral density (BMD) and increased risk of hip fracture [21, 22]. Furthermore, it is shown that undercarboxylated osteocalcin increases with age [23], and high levels are associated with both lower BMD and increased risk of hip fracture [24, 25]. In studies where calcium, vitamin D or vitamin K have been given as supplements to osteoporotic rats [26] or women [27], the results show a synergistic effect of giving calcium, vitamin D and vitamin K simultaneously. However, as far as we know, this has not been investigated as part of the diet in an intervention study.

Bone turnover is also reflected by different bone formation biomarkers such as bone alkaline phosphatase (BAP) [28], and bone resorption biomarkers, such as telopeptide (NTx) and deoxypyridinoline (DPYD) [29]. Anti-resorption osteoporosis treatment with bisphosphonates provides a decrease in bone turnover, both resorption and formation parameters, followed by an increase in BMD [30].

In most intervention studies where changes in BMD have been investigated as part of osteoporosis treatment, the intervention periods are at least around nine months [31, 32]. Thus, we aimed to have the focus on bone biomarkers and nutrition status where alterations previously have been detected within one month and more after osteoporosis drug treatment [33]. Therefore, the main aim of the present intervention trial was to investigate if intake of vitamin D enriched Atlantic salmon or vitamin D tablets decreased the bone turnover markers (urinary NTx, DPYD, s-BAP, and osteocalcin) compared to a low vitamin D intake. Secondary aim was to examine potential effects of increased intake of tailor-made Atlantic salmon on vitamin K status measured by s-undercarboxylated osteocalcin (GLU), s-carboxylated osteocalcin (GLA), and the GLU/GLA ratio. This also includes nutritional status measured by s-25-hyroxyvitamin D (25(OH)D), eicosapentaenoic acid (EPA) and docosahexaenoic acid (DHA), as well as BMD, body fat mass and lean mass.

## RESULTS

### Tailoring of farmed Atlantic salmon

**Figure 1:**
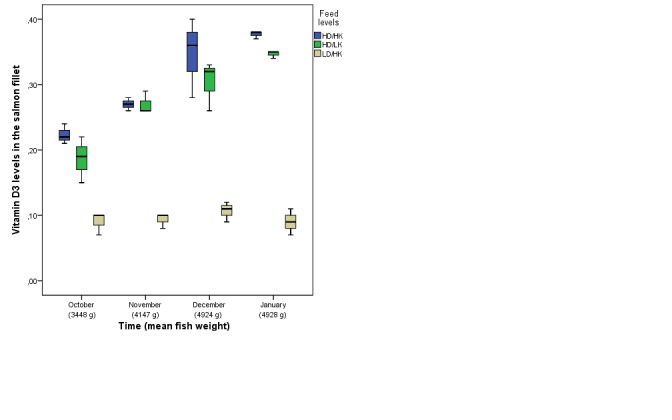
The different level of vitamin D(mg/kg)in the salmon fillet, time (October 2008 to January 2009) and mean salmon weight according to the feed levels/groups HD/HK, salmon fed high content of vitamin D_3_ and K_1_; HD/LK, salmon fed high content of vitamin D_3_ and low content of vitamin K_1_; LD/HK, salmon fed low content of vitamin D_3_ and high content of vitamin K_1_.

The different levels of vitamin D_3_ and K_1_ in the fish feed were reflected in the salmon fillet with increasing fish weight (Figure 1). The desired levels of vitamin D_3_ of at least 0.25 mg/kg were achieved according to the study design. There was also an increased level of vitamin K_1_ in the fillet according to the design.

### Characteristics of the participants

Baseline (pre-intervention) characteristics of the study population in total and by intervention group are given in Table 1. There were no significant differences between the groups for any of the included variables at pre-intervention. The median (interquartile range [IQR]) age of the 122 included women were 55.0 (5.0) years, and their body mass index (BMI) were 24.4 (5.0) kg/m^2^ at baseline.

**Table 1:** Baseline characteristics of all participants and by intervention group. Data are given as median (IQR) if not other is indicated

	Overall (n=122)	Tablets^a^ (n=30)	HD/HK^b^ (n=31)	LD/HK^c^ (n=30)	HD/LK^d^ (n=31)	*p*-value^e^
Demographics						
Age (year)	55.0 (5.0)	56.5 (7.0)	54.0 (6.8)	54.5 (4.3)	56.0 (5.5)	0.285
Body weight (kg)	68.2 (15.3)	67.7 (16.9)	73.1 (14.8)	68.8 (16.8)	65.5 (11.7)	0.204
BMI (kg/m^2^)	24.6 (5.0)	24.1 (6.6)	25.8 (5.1)	24.6 (4.8)	23.8 (3.1)	0.137
Current smoking, N (%)	15 (12.3)	3 (10.0)	4 (12.9)	2 (6.7)	6 (19.4)	0.407
Biological parameters						
Urinary NTx/Creatinine (mmol/l)	51.0 (29.8)	50.0 (21.8)	47.0 (41.0)	56.5 (31.5)	48.0 (27.0)	0.432
Urinary DPYD/Creatinine (mmol/l)	8.9 (2.7)	9.3 (2.2)	8.8 (2.5)	8.9 (3.4)	8.5 (3.4)	0.797
S-BAP (μg/l)	15.0 (7.1)	15.1 (11.0)	15.2 (5.7)	15.5 (9.9)	12.2 (6.5)	0.069
S-osteocalcin (ng/ml)	15.2 (7.2)	13.1 (7.4)	16.0 (6.7)	15.5 (8.1)	14.5 (8.2)	0.196
S-GLU (ng/ml)	4.1 (3.2)	4.2 (3.5)	4.4 (3.5)	3.9 (4.0)	3.4 (3.1)	0.216
S-GLA (ng/ml)	21.2 (11.4)	19.0 (11.3)	18.1 (10.6)	25.0 (13.0)	21.7 (12.6)	0.140
GLU/GLA ratio	0.206 (0.16)	0.19 (0.13)	0.27 (0.30)	0.22 (0.17)	0.17 (0.14)	0.251
S-25(OH)D (nmol/L)	75.4 (30.5)	77.1 (36.6)	74.2 (32.5)	74.4 (33.1)	74.4 (29.0)	0.916
S-PTH (pmol/l)	3.6 (2.6)	4.1 (2.4)	3.8 (1.6)	3.8 (1.7)	3.8 (1.4)	0.682
S-Creatinine (mmol/l)	5.9 (5.8)	5.9 (4.8)	6.7 (6.6)	5.2 (5.9)	6.6 (6.2)	0.564
n-3 EPA (mg/g RBC)	0.030 (0.030)	0.035 (0.023)	0.030 (0.020)	0.040 (0.015)	0.030 (0.030)	0.497
n-3 DHA (mg/g RBC)	0.105 (0.030)	0.110 (0.040)	0.100 (0.040)	0.110 (0.033)	0.100 (0.030)	0.111
Omega-3 index^f^	7.99 (2.59)	8.01 (3.21)	8.03 (2.21)	8.01 (2.60)	7.94 (2.80)	0.718
DXA-measurements						
Total body BMD (g/cm^2^)	1.06 (0.11)	1.06 (0.11)	1.05 (0.10)	1.05 (0.15)	1.05 (0.09)	0.947
Total body fat mass (%)	35.2 (8.1)	35.2 (8.1)	36.4 (5.6)	33.3 (7.3)	33.8 (5.2)	0.433
Total body fat mass (kg)	23.7 (8.9)	23.6 (11.4)	25.7 (8.2)	22.9 (9.4)	22.3 (7.4)	0.576
Total body lean mass (kg)	44.1 (5.5)	44.1 (5.5)	43.9 (7.4)	44.9 (6.2)	43.1 (7.9)	0.567
Daily dietary intake						
Total energy (KJ)	8666 (3631)	9288 (3158)	8282 (2945)	8628 (2819)	8739 (3523)	0.537
Protein (g)	92.1 (36.5)	96.6 (34.0)	90.4 (24.1)	92.4 (39.9)	89.2 (31.0)	0.742
Carbohydrate (g)	228.3 (99.6)	256.4 (85.9)	216.9 (78.3)	221.6 (96.2)	233.6 (117.2)	0.440
Total fat (g)	78.5 (41.5)	82.2 (51.7)	75.5 (39.1)	78.1 (32.2)	81.1 (42.9)	0.549
Saturated fat (g)	27.1 (15.4)	27.3 (13.9)	24.6 (12.8)	27.9 (14.1)	27.5 (17.0)	0.312
Monounsaturated fat (g)	26.8 (14.4)	27.7 (17.6)	26.0 (15.5)	26.6 (14.7)	27.4 (13.0)	0.649
Polyunsaturated fat (g)	15.5 (8.4)	16.3 (8.6)	15.4 (8.7)	14.6 (9.2)	15.7 (8.2)	0.770
Total Vitamin D (μg)	9.0 (10.9)	8.4 (8.1)	8.6 (8.4)	11.9 (13.9)	9.0 (16.1)	0.804
Vitamin D from food (μg)	5.8 (4.0)	6.4 (4.0)	6.3 (3.7)	5.0 (3.8)	5.8 (4.7)	0.393
Total Ca (mg)	974.5 (610.3)	1153.0 (595.0)	862.0 (575.0)	991.0 (667.0)	913.0 (685.0)	0.053
Ca from food (mg)	934.5 (552.5)	1066.0 (628.0)	862.0 (415.0)	979.0 (513.5)	913.0 (685.0)	0.619
Seafood as dinner (weekly)	1.5 (0.0)	1.5 (1.5)	1.5 (1.5)	1.5 (0.0)	1.5 (0.0)	0.553
Seafood as spread (weekly)	0.5 (1.4)	0.5 (1.2)	0.5 (1.2)	0.5 (1.4)	0.5 (1.0)	0.760
Fish oil supplements (weekly)	0.0 (0.5)	0.0 (0.0)	0.0 (0.4)	0.0 (5.5)	0.0 (7.0)	0.369

Abbreviations: 25(OH)D, 25-hyroxyvitamin D; BAP, bone alkaline phosphatase; BMC, bone mineral content; BMD, bone mineral density; BMI, body mass index; Ca, calcium; DPYD, deoxypyridinoline; DXA, dual X-ray absorptiometry; n-3 EPA+DHA, omega-3 eicosapentaenoic acids+docosahexaenoic acids; GLA, carboxylated osteocalcin; GLU, undercarboxylated osteocalcin; N, numbers; NTx, telopeptides; IQR, interquartile range; PTH, parathyroid hormone; RBC, red blood cells; s, serum.

a Tablets, intervention with vitamin D and calcium tablets;

b HD/HK, intervention with tailor made salmon with high content of vitamin D_3_ and K_1_;

c LD/HK, intervention with tailor made salmon with low content of vitamin D_3_ and high content of vitamin K_1_;

d HD/LK, intervention with tailor made salmon with high content of vitamin D_3_ and low content of vitamin K_1_.

e P-value are given with one way analysis of variance between the intervention groups for normal disturbed variables and Kruskal-Wallis test for skewed disturbed variables (PTH, BMI, energy intake, and all other kinds of dietary intake), and Chi-square test for the categorical variable current smoking.

f Sum %EPA + %DHA (% of sum of total fatty acids).

### Pre- to post-intervention results

#### Bone biomarkers

**Figure 2:**
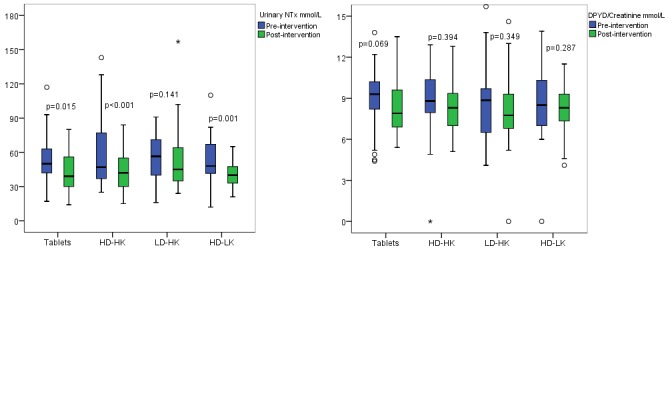
a, and b Pre-and post-intervention results of the bone resorption biomarker urinary N-telopeptides (NTx) (Figure 2a) and urinary deoxypyridinoline/creatinine (DPYD) (Figure 2b) within each intervention group. The horizontal line through the box represents the median. The lower boundary of the box is the 25^th^ percentile and the upper bounder is the 75^th^ percentile. The smallest and largest observed values within the distribution are represented by the horizontal at either end of the box. Tablets (n=30), intervention with vitamin D and calcium tablets; HD/HK (n=31), intervention with tailor made salmon with high content of vitamin D_3_ and K_1_; LD/HK (n=30), intervention with tailor made salmon with low content of vitamin D_3_ and high content of vitamin K_1_; HD/LK (n=31), intervention with tailor made salmon with high content of vitamin D_3_ and low content of vitamin K_1_. P between the groups for urinary NTx = 0.413 and urinary DPYD = 0.890.

The bone resorption biomarker urinary NTx decreased within all groups, except from within the low D_3_ + high K1 (LD/HK) group, but no significant group differences were observed (Table 2, Figure 2a). Urinary DPYD was unchanged within and between all groups (Table 2, Figure 2b).

**Table 2:** Changes in urinary and serum concentrations of bone turnover markers, vitamin D and fatty acids status during the intervention. Data are given as mean (SD)

	Tablets^a^ (n=30)			HD/HK^b^ (n=31)			LD/HK^c^ (n=30)			HD/LK^d^ (n=31)				
	Post	Delta^e^	*p*-value^f^	Post	Delta^e^	*p*-value^f^	Post	Delta^e^	*p*-value^f^	Post	Delta^e^	*p*-value^f^	*p*-value^g^	*p*-value^h^
NTx/ Creatinine (mmol/l)	47.6 (34.5)	−13.2 (27.9)	0.015	43.4 (17.5)	−15.8 (21.7)	<0.001	53.5 (28.1)	−6.2 (22.3)	0.141	40.8 (11.0)	−11.7 (22.8)	0.001	0.413	0.073
DPYD/ Creatinine (mmol/l)	8.3 (1.9)	−1.0 (2.8)	0.069	8.4 (1.8)	−0.5 (3.0)	0.394	8.1 (2.5)	−0.5 (3.0)	0.349	8.2 (1.8)	−0.5 (2.7)	0.287	0.890	0.958
Osteocalcin (ng/ml)	14.8 (7.1)	−0.9 (3.7)	0.199	14.4 (5.2)	−1.9 (3.5)	0.006	15.6 (7.0)	−2.1 (5.4)	0.038	14.4 (6.2)	0.4 (4.0)	0.598	0.084	0.300
GLU (ng/ml)	4.6 (3.2)	−0.3 (1.3)	0.190	3.6 (1.8)	−1.5^i^ (2.0)	<0.001	4.4 (2.6)	−0.7 (1.5)	0.026	3.6 (1.9)	−0.2 (1.6)	0.477	0.015	0.024
GLA (ng/ml)	18.8 (4.4)	−3.0 (7.6)	0.043	19.5 (5.3)	0.5 (7.3)	0.721	21.6 (6.3)	−2.3 (6.9)	0.076	18.1 (6.1)	−4.1^j^ (6.1)	0.001	0.070	0.050
GLU/GLA ratio	0.26 (0.23)	−0.03 (0.22)	0.510	0.19 (0.07)	−0.14^k^ (0.21)	0.001	0.21 (0.10)	−0.02 (0.12)	0.279	0.21 (0.13)	0.01 (0.14)	0.643	0.010	0.004
BAP (μg/l)	15.7 (9.0)	−1.6 (5.2)	0.103	15.4 (4.7)	0.2 (3.1)	0.756	17.3 (8.1)	−1.2 (4.1)	0.108	13.4 (5.3)	−0.7 (4.1)	0.347	0.374	0.556
25(OH)D (nmol/l)	85.4 (20.8)	13.7 (17.0)	<0.001	84.0 (15.6)	11.4 (16.0)	<0.001	69.7 (18.8)	−1.2^l^ (12.3)	0.590	86.8 (18.1)	12.1 (16.8)	<0.001	0.001	<0.001
EPA (mg/g)	0.03 (0.01)	−0.01^m^ (0.02)	<0.001	0.05 (0.01)	0.02 (0.02)	<0.001	0.05 (0.01)	0.01 (0.02)	0.038	0.05 (0.01)	0.01 (0.01)	0.001	<0.001	<0.001
DHA (mg/g)	0.09 (0.01)	−0.02^n^ (0.02)	<0.001	0.11 (0.02)	0.00 (0.02)	0.511	0.10 (0.01)	−0.01 (0.02)	0.026	0.09 (0.02)	0.00 (0.02)	0.205	0.001	<0.001
Omega-3 index^t^	6.0 (1.2)	−2.2^m^ (1.5)	<0.001	7.6 (1.1)	−0.7 (1.6)	0.056	7.7 (1.1)	−0.2 (1.3)	0.505	7.5 (1.8)	−0.3 (1.6)	0.362	<0.001	<0.001

Abbreviations: 25(OH)D, 25-hyroxyvitamin D in serum (S); BAP, bone alkaline phosphatase in s; DHA, omega-3 docosahexaenoic acids in red blood cells (RBC); DPYD, deoxypyridinoline in urin; EPA, omega-3 eicosapentaenoic acids in RBC; GLA, carboxylated osteocalcin; GLU, undercarboxylated osteocalcin; N, numbers; NTx, telopeptides in urin; SD, standard deviation.

a Tablets, intervention with vitamin D and calcium tablets;

b HD/HK, intervention with tailor made salmon with high content of vitamin D_3_ and K_1_;

c LD/HK, intervention with tailor made salmon with low content of vitamin D_3_ and high content of vitamin K_1_;

d HD/LK, intervention with tailor made salmon with high content of vitamin D_3_ and low content of vitamin K_1_.

e Mean (SD) unadjusted changes during intervention.

f P-value are given with Paired-samples t-test for comparison of individual pre- and post-intervention values.

g P-value are given with unadjusted General linear model.

h P-value are given with General linear model adjusted for the current pre-variable. Pairwise comparisons in General linear models with Bonferroni correction shows:

i p<0.01 for individual group comparison with the Tablets and HD/LK groups in unadjusted and adjusted analyses.

j p<0.05 for individual group comparison with the HD/HK and LD/HK group in adjusted analyses.

k p<0.05 for individual group comparison with the Tablets, LD/HK and HD/LK groups in unadjusted and adjusted analyses.

l p<0.001 for individual group comparison with the Tablets, HD/HK, and HD/LK groups in unadjusted and adjusted analyses.

m p<0.001 for individual group comparison with the HD/HK, LD/HK and HD/LK groups in unadjusted and adjusted analyses.

n p<0.05 for individual group comparison with the HD/HK, LD/HK and HD/LK groups in unadjusted and adjusted analyses.

t The content of EPA and DHA in RBC membranes expressed as percent of total fatty acids.

**Figure 3:**
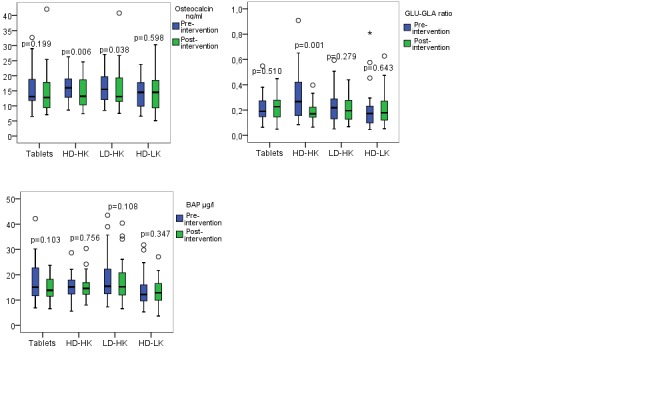
a, b, and c Pre-and post-intervention results of the bone formation biomarker serum (s)-osteocalcin (Figure 3a), the GLU/GLA ratio (Figure 3b), and s-one alkaline phosphatase (s-BAP) (Figure 3c) within each intervention group. The horizontal line through the box represents the median. The lower boundary of the box is the 25^th^ percentile and the upper bounder is the 75^th^ percentile. The smallest and largest observed values within the distribution are represented by the horizontal at either end of the box. Tablets (n=30), intervention with vitamin D and calcium tablets; HD/HK (n=31), intervention with tailor made salmon with high content of vitamin D_3_ and K_1_; LD/HK (n=30), intervention with tailor made salmon with low content of vitamin D_3_ and high content of vitamin K_1_; HD/LK (n=31), intervention with tailor made salmon with high content of vitamin D_3_ and low content of vitamin K_1_. P between the groups for s-osteocalcin = 0.084, for GLU/GLA ratio = 0.010, and for s-BAP = 0.374.

The bone matrix protein biomarker s-osteocalcin decreased within the HD/HK and LD/HK groups, but there were no differences between the groups (Table 2, Figure 3a). s-GLU decreased within the HD/HK and LD/HK groups, and significant differences between the groups were found with better outcome in the HD/HK group than the tablet (p=0.004), LD/HK (p=0.035) and high D_3_ + low K1 (HD/LK) groups (p=0.020). The vitamin K dependent s-GLA decreased within the tablet and HD/LK groups, and the outcome was better in the HD/LK group than the HD/HK (p=0.033) and LD/HK (p=0.018) groups (Table 2). GLU/GLA ratio decreased within the HD/HK group and differences showed better results in the HD/HK group compared to the tablet (p=0.001), LD/HK (p=0.025) and HD/LK (p=0.003) groups (Table 2, Figure 3b). S-BAP did not change within or between any of the groups from pre- to post-intervention (Table 2, Figure 3c).

### Nutritional status

**Figure 4:**
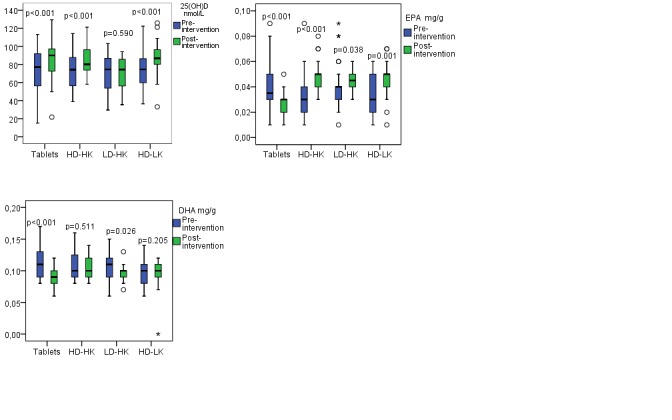
a, b and c Pre-and post-intervention results of serum 25-hydroxyvitamin D (s-25(OH)D) (Figure 4a), eicosapentaenoic acid (EPA) (Figure 4b) and docosahexaenoic acid (DHA) (Figure 4c) within each intervention group. The horizontal line through the box represents the median. The lower boundary of the box is the 25^th^ percentile and the upper bounder is the 75^th^ percentile. The smallest and largest observed values within the distribution are represented by the horizontal at either end of the box. Tablets (n=30), intervention with vitamin D and calcium tablets; HD/HK (n=31), intervention with tailor made salmon with high content of vitamin D_3_ and K_1_; LD/HK (n=30), intervention with tailor made salmon with low content of vitamin D_3_ and high content of vitamin K_1_; HD/LK (n=31), intervention with tailor made salmon with high content of vitamin D_3_ and low content of vitamin K_1_. P between the groups for s-25(OH)D = 0.001, EPA <0.001, and DHA = 0.001.

S-25(OH)D increased within all groups, except from in the LD/HK group from pre- to post-intervention, and the group differences were large (p<0.001) (Table 2, Figure 4a). EPA increased within all groups, except from in the tablet group, where decreased levels were found (p between the tablet and the other groups <0.001) (Table 2, Figure 4b). DHA decreased within the tablet and LD/HK groups, and was unchanged within the other groups, and significant differences between the groups were observed with better status in the HD/HK (p<0.001), LD/HK (p=0.032) and HD/LK groups (p=0.001) than in the tablet group (Table 2, Figure 4c). Furthermore, the omega-3 index decreased within the tablet group, whereas the results were unchanged in the other groups (p<0.001) (Table 2).

### Total fat mass and lean mass

Total body fat mass (%) decreased and total lean mass (kg) increased within the HD/HK group from pre- to post-intervention, but no significant differences between the groups were observed (Table 3). Total body BMD was also investigated but no significant difference within or between the intervention groups were observed (data not shown).

**Table 3:** Changes in body composition during the intervention. Data are given as mean (SD)

	Tablets^a^ (n=30)			HD/HK^b^ (n=31)			LD/HK^c^ (n=30)			HD/LK^d^ (n=31)				
	Post	Delta^e^	*p*-value^f^	Post	Delta^e^	*p*-value^f^	Post	Delta^e^	*p*-value^f^	Post	Delta^e^	*p*-value^f^	*p*-value^g^	*P*-value^h^
Total body fat mass (%)^k^	34.2 (6.5)	−0.3 (1.2)	0.115	34.5 (5.5)	−0.9^i^ (1.3)	0.001	33.2 (4.9)	−0.6 (1.8)	0.099	33.2 (5.0)	−0.1 (1.0)	0.605	0.141	0.184
Total body fat mass (kg)	24.8 (8.5)	−0.1 (4.2)	0.600	25.8 (8.0)	−0.4 (1.5)	0.181	23.8 (6.6)	−0.4 (1.8)	0.297	23.6 (7.5)	0.0 (1.0)	0.752	0.421	0.664
Total body lean mass (kg)	43.7 (4.4)	0.3 (1.0)	0.087	45.4 (5.5)	1.0_j_ (1.3)	<0.001	44.8 (4.9)	0.6 (5.7)	0.575	44.4 (6.1)	0.4 (1.0)	0.059	0.196	0.244

Abbreviations: SD, standard deviation.

a Tablets, intervention with vitamin D and calcium tablets;

b HD/HK, intervention with tailor made salmon with high content of vitamin D_3_ and K_1_;

c LD/HK, intervention with tailor made salmon with low content of vitamin D_3_ and high content of vitamin K_1_;

d HD/LK, intervention with tailor made salmon with high content of vitamin D_3_ and low content of vitamin K_1_.

e Mean (SD) unadjusted changes during intervention.

f P-value are given with Paired-samples t-test for comparison of individual pre- and post-intervention values.

g P-value are given with unadjusted General linear model.

h P-value are given with General linear model adjusted for the current pre-variable.

i p<0.05 for individual group comparison with the HD/LK group in unadjusted and adjusted analyses.

j p<0.05 for individual group comparison with the LD/HK group in unadjusted and adjusted analyses.

k Total body fat mass (%) = percent of soft tissue.

### Other analyses

S-bile acids, hydroxybutyrat, free fatty acids, glucose, glycerol, high-density lipoprotein (HDL), low-density lipoprotein (LDL) and total cholesterol, lactate, and triglycerides were also analyzed, but no significant differences within or between the groups from pre- to post-intervention were detected (data not shown).

### Eta squared calculations

Eta squared calculations for the analyses performed within the intervention groups as shown in Table 2 and 3, showed strong effect size [5] for all significant results from pre- to post-intervention (Eta squared >0.15).

### Compliance

In the tablet group, 90% of the women had less than seven missing days of supplements during the whole intervention (Table 4). In all salmon groups,92.4% of the participants reported that they had less than two missing study meals (Table 4). Thus, the compliance in all intervention groups was similar on the weekly basis.

**Table 4:** Reported compliance (missing days) of study meals and supplements in the different intervention groups. Data are given as numbers (%)

	Tablets^a^ (n=30)	HD/HK^b^ (n=31)	LD/HK^c^ (n=30)	HD/LK^d^ (n=31)
Calcigran (Nycoplus + Forte)				
No days	11 (36.7)	-	-	-
1-3 days	13 (43.3)	-	-	-
4-7 days	3 (10)	-	-	-
8-10 days	3 (10)	-	-	-
Study meals (salmon)^e^				
No days	-	26 (83.9)	24 (80)	22 (71)
1-2 days	-	3 (9.7)	2 (6.7)	8 (25.8)
3-5 days	-	2 (6.5)	4 (13.3)	1 (3.2)
Calcium (Weifa)^e^				
No days	-	16 (51.6)	10 (33.3)	14 (45.2)
1-3 days	-	7 (22.6)	7 (23.3)	10 (32.3)
4-7 days	-	6 (19.4)	7 (23.3)	5 (16.1)
8-10 days	-	1 (3.2)	6 (20)	0
11-14 days	-	1 (3.2)	0	2 (6.5)

a Tablets, intervention with vitamin D and calcium tablets;

b HD/HK, intervention with tailor made salmon with high content of vitamin D_3_ and K_1_;

c LD/HK, intervention with tailor made salmon with low content of vitamin D_3_ and high content of vitamin K_1_;

d HD/LK, intervention with tailor made salmon with high content of vitamin D_3_ and low content of vitamin K_1_.

e Chi-square test for comparison between the salmon groups separately for study meals and calcium showed p>0.05.

In addition, 100% of the participants in the HD/HK group, 96.7% in the LD/HK group and 90.3% in the HD/LK group consumed always the whole portion of salmon in every study meal. Four participants (one in the LD/HK group and three in the HD/LK group) consumed usually the whole portion of salmon. There was no significant differences between the groups (p<0.05).

### Dietary patterns

Ten (8.2%) of the participants reported changes due to dietary patterns, other than the intervention itself. Reasons for this were as following: four (one in each of the four intervention groups) reported that they started on a diet to reduce weight, three (one in each of the salmon groups) reduced intake of sweets, two (one in the LD/HK group and one in the HD/LK group) reported reduced intake of bread. In addition, one woman in the HD/HK group started on iron supplements. No significant differences between the groups were observed (p>0.05).

During the intervention, 60% of the women in the tablet group ate lean seafood ≥ two times the week, which was more often than the women in the HD/HK (29%), LD/HK (16.7%), and HD/LK (41.9%) groups (p=0.004).

### Side effects of Vitamin D tablets, Calcigran forte and calcium supplements

Side effects of vitamin D tablets and calcium supplements are shown in Table 5. The most common side effect was stomach upsets (abdominal pain, nausea, bloating, and constipation), and 12 (38.7%) of the participants in the HD/LK group reported stomach upsets often/always. Other side effects were headache and “tasted bad” (Table 5). Furthermore, two participants in the tablet group reported dry mouth often as a side effect. No participants reported side effects like tiredness. In addition, two participants (one in each of the HD/HK and HD/LK group) stopped using calcium supplements because of side effects.

**Table 5:** Reported side effects of vitamin D and calcium supplements. Data are given as numbers (%)

	Overall (n=122)	Tablets^a^ (n=30)	HD/HK^b^ (n=31)	LD/HK^c^ (n=30)	HD/LK^d^ (n=31)
Stomach upsets^e^					
Never	53 (43.4)	17 (56.7)	15 (48.4)	16 (53.3)	5 (16.1)
Rarely	17 (13.9)	0	5 (16.1)	6 (20.0)	6 (19.4)
Sometimes	29 (23.8)	10 (33.3)	9 (29.0)	2 (6.7)	8 (25.8)
Often	18 (14.8)	2 (6.7)	2 (6.5)	6 (20.0)	8 (25.8)
Always	5 (4.1)	1 (3.3)	0	0	4 (12.9)
Tasted bad					
Never	107 (87.7)	27 (90.0)	29 (93.5)	23 (76.7)	28 (90.3)
Rarely	3 (2.5)	1 (3.3)	0	1 (3.3)	1 (3.2)
Sometimes	1 (0.8)	0	0	1 (3.3)	0
Often	4 (3.3)	1 (3.3)	0	2 (6.7)	1 (3.2)
Always	7 (5.7)	1 (3.3)	2 (6.5)	3 (10.0)	1 (3.2)
Headache					
Never	121 (99.2)	29 (96.7)	31 (100)	30 (100)	31 (100)
Rarely	0	0	0	0	0
Sometimes	0	0	0	0	0
Often	1 (0.8)	1 (3.3)	0	0	0
Always	0	0	0	0	0

a Tablets, intervention with vitamin D and calcium tablets;

b HD/HK, intervention with tailor made salmon with high content of vitamin D_3_ and K_1_;

c LD/HK, intervention with tailor made salmon with low content of vitamin D_3_ and high content of vitamin K_1_;

d HD/LK, intervention with tailor made salmon with high content of vitamin D_3_ and low content of vitamin K_1_.

e Stomach upsets included abdominal pain, nausea, bloating, and constipation.

f Chi-square test for comparison between the intervention groups separately for stomach upsets, tasted bad and headache showed p>0.05.

## DISCUSSION

In the present study, Atlantic salmon fillet was successfully tailored yielding enriched levels of vitamin D and vitamin K. Earlier studies with smaller salmon (first-feeding and smolt) have also demonstrated increased levels of vitamin D in whole salmon [34] and salmon tissues [35] due to increased dietary level. However, this is the first time it has been shown in commercially sized fish.

Positive effects of intake of tailor-made Atlantic salmon on several of the investigated bone formation and resorption markers in healthy postmenopausal women were observed. Especially the HD/HK group had good outcomes on more of these parameters, including urinary NTx, s-osteocalcin, s-GLU and the GLU/GLA ratio. We might speculate if our findings are a result of a synergy effect by giving calcium, vitamin D, and K simultaneously as earlier observed in supplemental studies. Vitamin K is important for activating osteocalcin [16], and carboxylation of osteocalcin is a marker of vitamin K status [17, 18]. Most of the documentations of vitamin K related to bone health is based on studies with vitamin K_2_ [27, 36]. However, even though the documentations of similar effects of vitamin K_1_ is not as strong as K_2_, a combination of low serum levels of vitamins K_1_ and D have been associated with increased risk of hip fracture in elderly [37]. Both low levels and intakes of vitamin K_1_ have been associated with osteoporosis [19–22], and our findings indicate that vitamin K_1_ has a positive effect as well.

Our participants in the different groups had adequate levels of s-25(OH)D at baseline, but levels above 75 nmol/L are recommended to maximize vitamin D's beneficial effects for health [38]. However, it is still not documented in randomized controlled studies if levels above 75 nmol/L can improve BMD, decrease fracture risk and bone turnover markers.

We found increased levels of s-25(OH)D in all groups from pre- to post-intervention, except in the LD/HK group, which indicate that intake of standard Atlantic salmon alone is not enough to improve vitamin D status. The content of vitamin D_3_ in fish fillets reflects the fish feed vitamin D_3_ content [34]. The salmon served in our LD/HK group contained an average of 0.09 mg vitamin D_3_ per kg fillet, which is equivalent with data on commercial farmed salmon fillet from 2006 (0.08 mg/kg) [4]. However, with steadily less marine ingredients and more plant based material, vitamin D_3_ content in fish feed has decreased further and data from 2012 show an average value of 0.06 mg/kg fillet [4]. In addition, the increments in vitamin D in the current study were as large in the fish groups (HD/HK and HD/LK) as in the tablet group. Even though most of the participants had sufficient vitamin D status at enrollment, two servings/week with tailor-made Atlantic salmon fed with high vitamin D_3_ (0.35-0.38 mg/kg/fillet) further increased their vitamin D status. We did not find any difference in fish or vitamin D intake at baseline in our participants, thus the follow-up results seem to be reliable. Except for a significant higher intake of lean fish in the tablet group, there was no other differences in dietary pattern between the intervention groups. Lean fish does not contribute with vitamin D.

All groups eating salmon had increased levels of EPA from pre- to post-intervention. DHA was unchanged or even decreased in the LD/HK group. These findings can probably be explained by the fact that the amount of DHA in the fish fillet was not high enough to give any further increase. In the tablet group, the participants had decreased levels of both EPA and DHA.

Our results show that two meals/week with tailor-made salmon seems to maintain the omega-3 index; even though the DHA was relatively low in the current salmon fillet. The omega-3 index has been used to predict coronary heart disease in previous studies, and based on these findings an omega-3 index ≥ 8% is related to greatest protection, and ≤ 4% to least protection [39]. All our intervention groups had an omega-3 index median of approximately 8% at baseline, and the fish groups preserved this level at post-intervention, however the tablet group had a relatively large reduction, and at follow-up, the level was 5.7%. These findings shows that lean fish alone, even with a higher intake compared to the salmon groups, is not enough to keep the level of EPA and DHA in RBC.

Generally, animal studies support the favorable effect of omega-3 LC-PUFAs on bone health parameters [40]. In humans, few studies have been conducted, but positive effects of omega-3 LC-PUFAs primarily given as fish oil or capsules on bone biomarkers and BMD have been observed [41], but not in all studies [42]. In the Framingham Osteoporosis Study, high intake of fatty fish and particularly dark fish with high levels of omega-3 LC-PUFA was associated with protective effects on BMD as exposed over a period of four years [43]. However, we did not find any differences in BMD between the intervention groups, and the reason may be the relatively short intervention period.

The HD/HK group also had a small significant reduction in fat mass (%) and increased lean mass from pre- to post-intervention. Omega-3 LC-PUFAs have shown to be a useful therapeutic agent for sarcopenia [44], which is closely related to osteoporosis [45], but not relevant for the included women in this study. Anyway, the ability of the skeletal muscle to use amino acids to build constitutive proteins is gradually lost with age and this is partly due to decline in skeletal muscle insulin sensitivity. Omega-3 LC-PUFA can improve insulin-mediated glucose metabolism in insulin-resistant states [46]. It should also be mentioned that not only omega-3 LC-PUFAs, but also adequate vitamin D status is important when it comes to muscle strength and risk of falls, and thus fractures in elderly [47, 48]. Therefore, tailor-made salmon might also be a good source in the diet for prevention of falls and fractures.

The main strengths of this study are the randomized intervention design where we had the opportunity to enrich the fish feed with different content of vitamin D_3_ and K_1_. The participants in the fish groups were blinded. In addition, the compliance was good. However, our study has some limitations. The trial was performed in a relatively young, healthy sample of postmenopausal women aged 50-64 years. Reduced bone health is more common in older women, but this age span was chosen because these women are especially prone to develop osteoporosis because of estrogen decline. From a clinical point of view, it is important to search for clinical effects that can prevent osteoporosis. In the next step, hopefully this can increase quality of life and reduce the socioeconomic cost of having osteoporotic patients in hospital. Next to age and female gender, BMD is the strongest predictor of osteoporotic fractures [49], and femoral neck BMD is the recommended reference area for description of osteoporosis [50]. We had BMD measurements of total body only, but as the intervention period was relatively short, we did not expect any large changes in the participants BMD.

In conclusions, increased intake of tailor-made salmon containing high levels of vitamin D_3_ (0.35-0.38 mg/kg/fillet), and supplements with the same weekly contribution had a positive influence on bone health as measured by bone biomarkers in postmenopausal women. Consequently, an increased level of vitamin D_3_ at least to original level in feed for salmonids will contribute to an improved vitamin D_3_ status and may have a positive effect on human bone health.

## MATERIALS AND METHODS

### Ethics statement

Informed consent was obtained from all participants, and the study was approved by the Regional Committee for Research Ethics West (252.07 and 2010/605-3), and the Norwegian Social Science Data Service. The study was registered at ClinicalTrials.gov (NCT02615301).

### Sample size and power calculation

Power calculation revealed that a minimum of 30 subjects should be included in each intervention group. The calculation was based on vitamin D tablets and an estimated change of 10% from pre- to post-intervention. Strength was set to 80%. It was also taken into account a measurement uncertainty of 10% on *high-performance liquid chromatography* (HPLC) used to analyse vitamin D metabolites, and an assumed dropout rate of approximately 20%.

### Study population

**Figure 5:**
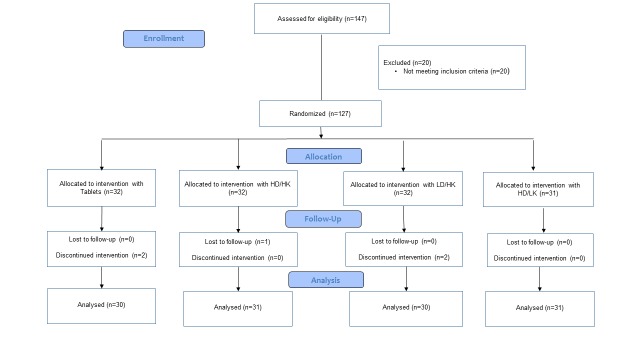
Flow-chart showing the study population Tablets, intervention with vitamin D and calcium tablets; HD/HK, intervention with tailor made salmon with high content of vitamin D_3_ and K_1_; LD/HK, intervention with tailor made salmon with low content of vitamin D_3_ and high content of vitamin K_1_; HD/LK, intervention with tailor made salmon with high content of vitamin D_3_ and low content of vitamin K_1._

The participants in the current randomized intervention trial were recruited through a local newspaper in Bergen, Norway in January 2009, and 147 women were interested to participate in the study (Figure 5). Of these, 20 women were excluded after telephone interview because they did not meet the inclusion criteria. Inclusion criteria comprised postmenopausal women, Caucasian ethnicity, age range 50-65 years and having postmenopausal age of at least one year. To minimize any confounding effects on bone health parameters like osteocalcin and BMD, subjects with the following conditions were excluded; osteoporotic fracture, medical treatment for osteoporosis, warfarin treatment, creatinine above or below normal range, hypervitaminosis D, malabsorption syndrome, inflammatory bowel disease, or inflammatory rheumatic diseases. In addition, women who had planned to go away on holiday during the intervention could not participate. No participants were excluded after the physical examination. The 127 (86.4%) women were individually randomized into four intervention groups: three salmon groups and one tablet group. The salmon had three different vitamin D_3_/vitamin K_1_ combinations: high D_3_ + high K_1_ (HD/HK), low D_3_ + high K_1_ (LD/HK), or high D_3_ + low K_1_ (HD/LK). Four women did not complete the intervention period, and one woman was excluded from the analyses after the intervention because of missing blood samples. Thus, 122 (83%) women were included in the final study sample (Figure 5).

### Tailor-made Atlantic salmon

The Atlantic salmon (*Salmo salar L.)* was produced at GIFAS (Gildeskål Research station, Inndyr, Norway). The fish were kept in net pens (5x5x5 m) in the sea and fed a dry, extruded feed for four months from an initial body weight of two kg. The feed (produced at Skretting ARC Feed Technology Plant) contained 38% crude protein and 34% crude fat, in accordance with commercial diets for this size of salmon. The dry matter of the feed was 94.6-94.8%. The feed contained three different levels of vitamin D_3_ (cholecalciferol) and vitamin K_1_ (phylloquinone), in three different combinations: HD/HK 2.9 and 4.3 mg/kg, respectively, LD/HK 0.23 and 4.7 mg/kg, HD/LK 2.6 and <0.001 mg/kg. The fish were harvested at about 4.5 kg and slaughtered at Fiskekroken AS, Sørarnøy, Norway. The fish were transported by car *pre rigor,* and processed into skin and boneless portions (150 g), vacuum packed, and frozen by Rex Star Seafood (Tysnes, Norway). The fish were transported frozen to National Institute of Nutrition and Seafood Research (NIFES), Bergen, Norway for storage at −30°C until use.

### Experimental groups

Prior to randomization, the vitamin D status of the participants was assessed. Thereafter the randomization process was run electronically until there were no significant differences in mean vitamin D status between the four groups. The randomization was done according to procedures recommended by the Norwegian Institute of Public Health [51]. Researchers at NIFES did the randomization, and technicians at Center of Clinical Trials, Bergen, Norway did the enrolling and assigning of participants to the study. The participants in the fish groups were blinded to dose of vitamin D3 and K1 in their respective study group. In addition, the intervention groups were coded and researchers and technicians at NIFES were blinded until statistical analyses were done. The fish groups were provided with farmed Atlantic salmon in portions of 150 grams and hand-outs with dinner recipes. They were instructed to have salmon for dinner two times per week for a period of 12 weeks. The study was run from February to May to minimize the effect of UV exposure on vitamin D status. The control group was provided with two different types of tablets; Calcigran Forte® containing 400 IE vitamin D and 500 mg calcium, and Nycoplus Calcigran® containing 200 IE vitamin D and 500 mg calcium, and was instructed to take one tablet of each type every day during the intervention. An overview of the weekly intake of vitamin D3 and K1 in the different intervention groups are given in Table 6. They were informed to avoid oily fish in the same period, but had no restrictions on lean fish. The fish groups also received calcium supplements (1000 mg/d) but without vitamin D (Weifa-Kalsium®). The participants registered the consumed amount of salmon and tablets in a diary. The total fat- and protein content of the salmon was 14.5 and 19.5 g per 100 grams fillet, respectively. The intake of EPA+DHA in a portion (150 g) of Atlantic salmon was 2.8 g. The levels of vitamin D_3_ and K_1_ in the salmon for the HD/HK group was mean 0.38 (SD 0.01) and 0.54 (0.06) mg/kg, respectively, for the LD/HK group 0.09 (0.02) and 0.58 (0.10) mg/kg, and for the HD/LK group 0.35 (0.01) and 0.03 (0.00) mg/kg (Table 6).

**Table 6:** Weekly intake of vitamin D_3_ and K_1_ in the different intervention groups

	Vitamin D_3_ (μg)	Vitamin K_1_ (μg)
Tablets^a^	105	0
HD/HK^b^	114	162
LD/HK^c^	27	162
HD/LK^d^	105	9.6

a Tablets, intervention with vitamin D and calcium tablets;

b HD/HK, intervention with tailor made salmon with high content of vitamin D_3_ and K_1_;

c LD/HK, intervention with tailor made salmon with low content of vitamin D_3_ and high content of vitamin K_1_;

d HD/LK, intervention with tailor made salmon with high content of vitamin D_3_ and low content of vitamin K_1_.

The content of several undesirables substances were also determined in the Atlantic salmon. The level of mercury was 0.03 mg/kg whereas the level of dioxins and dioxin-like PCBs was 0.7 ng TEQ/kg, which are both far below the EUs upper limits of 0.5 mg/kg and 6.5 ng TEQ/kg in fish, respectively. Taking into account the amount of salmon consumed per week, the intake of dioxin and dioxin-like PCBs per week represents 21% of the tolerable weekly intake (TWI) in a person weighted 70 kg [52]. For persons with higher body weight, salmon will contribute with a correspondingly lower percentage of TWI.

### Pre- and post-intervention measurements

#### Analytical procedures

Venous blood samples were drawn from non-fasting participants, collected into tubes, and separated through centrifugation (10 minutes, 3000 g, 20°C). Red blood cells (RBC) were used for analyses of fatty acid profile, whereas serum samples were used for other parameters. Second morning urine samples were collected from each participant, and RBC, serum and urine samples were kept frozen at −80 °C until analysis.

BAP (Quidel Corporation, San Diego, CA, USA), s-osteocalcin (Nordic Bioscience Diagnostics, Herlev, Denmark), s-GLU and s-GLA (ng/ml) were analysed at the Hormone Laboratory, Haukeland University Hospital, Bergen, Norway by enzyme-linked immunosorbent assays as described in Emaus et al [53].

Vitamin D status was assessed by measuring s-25(OH)D levels. Ultra performance liquid chromatography with tandem mass spectrometry detection (LC-MS/MS) was applied for the analysis. The applied experimental method was developed at NIFES based on the method described by Kissmeyer et al [54].

Fatty acids composition of total RBC was determined at NIFES by ultrafast gas chromatographic (UFGC) (Thermo Electron Corporation, Massachusetts, USA) developed by Araujo et al [55]. The fatty acids composition was calculated using an integrator (Chromeleon 6.80, Dionex Corporation, California, USA) connected to the UFGC and identification ascertained by standard mixtures of methyl esters (Nu-Chek, Minnesota, USA). The RBC analyses are described in details by Markhus et al [56]. The omega-3 index is the percent of EPA and DHA in RBC membranes expressed as percent of total fatty acids [39].

Urinary creatinine was assessed according to routine methodology at NIFES as described by Julshamn et al [57]. Urinary creatinine was measured spectrophotometerically (Technicon RA 1000) together with a standard reference material (Bayer Testpoint^TM^ assayed chemistry control 1).

S-bile acids, hydroxybutyrat, free fatty acids, glucose, glycerol, HDL, LDL and total cholesterol, lactate, triglycerides were assessed at NIFES according to Aadland et al [58].

Serum PTH, Urinary NTx/creatinine and urinary DPYD/creatinine were assessed according to routine methodology at Aker University Hospital, Oslo, Norway.

### Bone mineral density, body composition and body mass index

The total body BMD, total body soft tissue composition (fat mass and lean mass) measurements, and weight were performed using dual-energy X-ray absorptiometry (DXA) on a stationary fan beam densitometer (Hologic; QDR4500A) at the Center of Clinical Trials. All measurements were done on the same DXA machine by one trained technician. Percentage fat mass was calculated as fat mass in kilogram in percent of total body weight in kilogram. BMI was calculated as weight (kg) divided by height squared (m^2^). Height was measured by a big ruler.

### Nutrition, physical activity, smoking and side effects

Information about nutrition, physical activity and smoking were obtained at the same time as the DXA-assessments and sampling of blood and urine.

Dietary habits the last three months were determined using two validated food frequency questionnaires (FFQ), “What do you eat?” [59] and “Seafood intake” [60]. The completion of the questionnaires was done by the participants with a nutritionist available nearby for possible explanations to questions the participants might have. One of the FFQs (“What do you eat”) was read optically, and a database and a software system (KBS software, version 3.2; University of Oslo, Norway) developed at the Department of Nutrition, University of Oslo, was used to calculate the dietary intake. Total energy intake (EI, kcal), and intake of macronutrients (proteins, lipids and carbohydrates) and micronutrients (minerals and vitamins) were estimated and compared with dietary reference intakes (RDIs). We previously calculated a seafood index which was used for presentation of the pre-intervention results of “seafood dinner”, “seafood other than dinner”, and “fish oil supplements”. These calculations are described in details elsewhere [56].

Information of physical activity and current smoking were obtained from the “Seafood intake” questionnaire. However, data on physical activity is not included (no statistical differences were observed between the groups).

In addition, the participants reported changes due to dietary eating patterns during the intervention period retrospectively, as well as registration of side effects such as stomach upsets, including abdominal pain, nausea, bloating, and constipation, and headache, “tasted bad”, dry mouth as well as tiredness were registered in a self-reported questionnaire which the participants filled out after the intervention period.

### Statistical analyses

Continuous variables are expressed as median with IQR, and mean with standard deviation (SD), and the categorical variable current smoking as numbers and percentages. At pre-intervention, one-way analysis of variance was used for continuous normal distributed variables for comparisons between the intervention groups. Kruskal-Wallis test was used for continuous skewed disturbed variables (PTH, BMI, energy intake, and all other kind of food intake as presented, and Chi-square test for the categorical variable current smoking for comparisons between the intervention groups at pre-intervention, as well as for comparison of compliance, dietary patterns and side effects between the intervention groups. Paired-samples t-test was used for calculations of pre-and post-intervention results within each group. In addition, the effect sizes (Eta squared) for the paired-samples t-test results were calculated. General linear model analyses without and with adjustments for the current pre-variable were performed for the delta results in comparison between the intervention groups, and pairwise group comparisons with Bonferroni correction were done if overall p-value was significant. Urinary NTx, DPYD, s-osteocalcin, GLU/GLA ratio, s-25(OH)D, EPA and DHA were also presented using box-plots, and in addition to median, the 25^th^ and the 75^th^ percentile were given. All analyses covers the 122 included participants.

Two-tailed *p* values <0.05 were considered statistically significant. The analyses were performed using the Statistical Package for the Social Sciences (SPSS) for windows (IBM SPSS Statistics 23, Chicago, IL, USA, www.spss.com).
